# Urologische Ausbildung und Arbeitsattraktivität für die Generation Z – ein Rapid Review

**DOI:** 10.1007/s00120-025-02742-y

**Published:** 2025-12-09

**Authors:** Stefanie Cermak, Andreas Wencker, Lujza Brunaiova, Lukas Koneval, Beat Roth, Laila Schneidewind

**Affiliations:** 1 Zentrum für Urologie und Nephrologie, Bern, Schweiz; 2 Kantonsspital Aarau, Aarau, Schweiz; 3 Universitätsklinik für Urologie, Universität Bern, Inselspital Bern, Wilhelm-Fabry-Haus, Freiburgstr. 37, 3010 Bern, Schweiz

**Keywords:** Facharztausbildung, Urologische Ausbildung, Arbeitsplatzattraktivität, Work-Life-Balance, Evidenz, Generation Z, Residency, Urological education, Job attractiveness, Work-life balance, Evidence, Generation Z

## Abstract

**Hintergrund:**

Es ist bekannt, dass die Generation Z andere Bedürfnisse und Erwartungen in Hinblick auf die Arbeitswelt haben als frühere Generationen.

**Fragestellung:**

Es wird eine schnelle Evidenzanalyse zur Generation Z in der ärztlichen Ausbildung durchgeführt. Das primäre Ziel war es, die Charakteristika dieser Generation im Kontext der medizinischen Ausbildung und des Arbeitsmarktes zu beschreiben.

**Material und Methoden:**

Die Literatursuche wurde in MEDLINE via PubMed für den Zeitraum Januar 2000 bis zum Datum der letzten Suche (07. August 2025) durchgeführt. Für die Evidenzsynthese wurden lediglich Originalarbeiten und systematische Reviews genutzt.

**Ergebnisse:**

Die primäre Literatursuche ergab 1188 Treffer, schließlich konnten lediglich 3 Arbeiten eingeschlossen werden, davon 2 Umfragen und eine systematische Übersichtsarbeit. Generation Z misst der Arbeitszufriedenheit einen hohen Stellenwert bei und wünscht langfristige Perspektiven. Sie ist bereit zum Wohle anderer mit hohem Maß an Ethik zu arbeiten und dabei gleichzeitig bereit Herausforderungen anzunehmen. Dabei hat sie ein hohes Verantwortungsbewusstsein und Engagement für langfristige Ziele. Der Wunsch nach Feedback und sofortiger Rückmeldung bzw. proaktiver Kommunikation seitens der Vorgesetzten ist essentiell. Digitale Lösungen für Lernen, Arbeit und Kommunikation werden bevorzugt.

**Schlussfolgerung:**

Für die Generation Z muss ein sicheres Arbeitsumfeld mit langfristigen und nachhaltigen Perspektiven geschaffen werden. Die Integration moderner Technologien in den Arbeitsalltag ist von enormer Bedeutung. Leider ist die aktuelle Evidenzlage insbesondere in der Urologie begrenzt.

## Abstract (en)

**Background:**

It is well known that Generation Z has different needs and expectations with regard to the world of work to previous generations.

**Objective:**

A rapid evidence analysis addressing Generation Z in medical education was conducted. The primary objective was to describe the characteristics of this generation in the context of medical education and the labor market.

**Materials and methods:**

The literature search was conducted in MEDLINE via PubMed for the period from January 2000 to the date of the last search (August 7, 2025). Only original articles and systematic reviews were used for the evidence synthesis.

**Results:**

The primary literature search yielded 1188 hits, but ultimately, only three studies could be included, two of which were surveys and one a systematic review. Generation Z attaches great importance to job satisfaction and wants long-term prospects. They are willing to work with a high degree of ethics for the benefit of others, while at the same time being prepared to take on challenges. They have a strong sense of responsibility and commitment to long-term goals. They have an essential desire for feedback and immediate response or proactive communication from supervisors. Digital solutions for learning, work, and communication are preferred.

**Conclusion:**

A secure working environment with long-term and sustainable prospects must be created for Generation Z. The integration of modern technologies into everyday working life is of enormous importance. Unfortunately, the current evidence base is limited, particularly in urology.

## Hintergrund und Fragestellung

Angehörige der Generation Z wurden zwischen 1996 und 2015 geboren und sind erstmalig 2013 in höhere Bildungswege eingetreten. Sie ist die erste Generation, die in einem vollständigen digitalen Umfeld aufgewachsen ist [1]. Daher werden auch wir Urologen zukünftig mit Medizinstudierenden sowie Facharztkandidaten dieser Generation arbeiten. Zusätzlich gibt es eine zunehmende Anzahl von Veröffentlichungen über Studierende der Generation Z, die zeigen, dass sie andere Bedürfnisse und Erwartungen haben als frühere Generationen. Vor allem sind diese Studierenden stark auf Informationstechnologie angewiesen und bevorzugen es, selbstständig und in ihrem eigenen Tempo zu lernen und zu arbeiten [1, 2]. Dies kann für die Zusammenarbeit als auch für das Lernumfeld Vor- und Nachteile mit sich bringen.

Die klinische Ausbildung in Bezug auf Studierende der Generation Z ist ein besonderer Problembereich

Weiterhin stellt die klinische Ausbildung in Bezug auf Studierende der Generation Z einen besonderen Problembereich dar. Es gibt bereits einige Hinweise aus der Kohorte der Krankenpflegeschüler, dass sie ihre klinische Ausbildung aufgrund unzureichender Zeit in der Klinik und Mängeln im klinischen Lernumfeld als unzureichend empfanden [1, 3–5]. Leider gibt es bisher nur spärliche Daten zur ärztlichen Ausbildung der Generation Z, insbesondere zur Facharztausbildung in der Urologie.

Aufgrund des demographischen Wandels sowie der Nachwuchsproblematik sind wir Urologen allerdings gezwungen, die ärztliche und fachärztliche Ausbildung zu verbessern, um die hoch qualitative urologische Versorgung nach aktueller Evidenzlage zu gewährleisten sowie die Arbeitsplatzattraktivität zu steigern.

Konsequenterweise führten wir eine schnelle Evidenzanalyse zur Generation Z in der ärztlichen Ausbildung durch. Das primäre Ziel war es, die Charakteristika dieser Generation im Kontext der medizinischen Ausbildung und des Arbeitsmarktes zu beschreiben. Sekundär sollte untersucht werden, welche Implikationen sich hieraus für die Gestaltung der Facharztausbildung sowie für die Steigerung der Attraktivität ärztlicher Arbeitsplätze ergeben.

## Methodik

Es wurde eine schnelle Evidenzanalyse mit Literaturrecherche in MEDLINE via PubMed für den Zeitraum Januar 2000 bis zum Datum der letzten Suche (07. August 2025) durchgeführt [6]. Anzumerken ist, dass bei der Methodik der schnellen Evidenzanalyse lediglich eine Datenbank verwendet wird. Als Suchbegriffe wurden die Begriffe „generation Z“, „surgical education“ und „urology“ sowie aller möglichen Kombinationen dieser drei Begriffe verwendet. Für die Evidenzsynthese wurden lediglich Originalarbeiten und systematische Reviews genutzt, Fallberichte sowie narrative Übersichtsarbeiten wurden nicht berücksichtig. Weiterhin wurde ausschließlich Untersuchungen eingeschlossen, die die ärztliche Situation thematisieren.

Bezüglich der Sprache wurden nur englische und deutsche Arbeiten verwendet. Der primäre Endpunkt dieser Arbeit ist die Deskription der aktuellen Situation in der ärztlichen Ausbildung und am Arbeitsplatz für die Generation Z, insbesondere für das Fachgebiet Urologie, falls Daten hierzu verfügbar sind. Sekundäre Endpunkte stellen Implikationen für die Verbesserung der ärztlichen Ausbildung sowie für die Steigerung der Arbeitsplatzattraktivität für die Generation Z dar. Weiterhin wurden die PRISMA-Leitlinien („preferred reporting items for systematic reviews“) zur Berichterstattung systematischer Übersichtsarbeiten angewandt [7]. Auf die Qualitätsbewertung der inkludierten Untersuchungen wurde aufgrund der zu erwartenden Heterogenität verzichtet.

## Ergebnisse

**Abb. 1:**
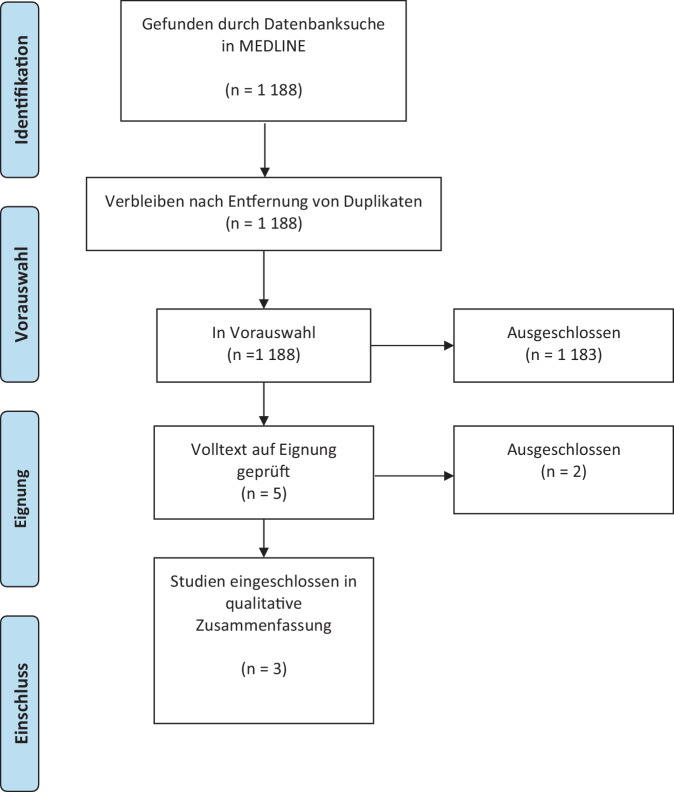
PRISMA-Flussdiagramm („preferred reporting items for systematic reviews“)

Die primäre Literatursuche ergab 1188 Treffer, schließlich konnten lediglich 3 Arbeiten eingeschlossen werden, davon 2 Umfragen und eine systematische Übersichtsarbeit (Abb. 1: PRISMA-Flussdiagramm). Die Tab. 1 gibt einen Überblick über die eingeschlossenen Studien sowie deren Charakteristika.

**Tab. 1:** Charakteristika und Hauptergebnisse der inkludierten Studien (*n* = 3)

Referenz	Studiendesign/Methodik	Studienland/Lokalisation	Hauptergebnisse	Implikationen für die ärztliche Ausbildung und Arbeitsplatzattraktivität	Schlussfolgerungen der Autoren
*Giunta et al. (2025; *[8])	Umfrage	Deutschland, Finnland, Frankreich, Großbritannien, Italien, Spanien, Türkei, USA	Die wichtigsten Ergebnisse zeigen einen Generationswechsel in der Einstellung zum Berufsleben, wobei der Schwerpunkt auf digitaler Innovation, Work-Life-Balance und der Interaktion mit sozialen Medien liegt	Fortschritte im Bereich des digitalen Lernens und der simulationsbasierten Ausbildung wurden als Chancen zur Förderung der medizinischen Ausbildung identifiziert	Der Eintritt der Generation Z in die plastische Chirurgie stellt einen möglichen Wendepunkt dar, um traditionelle Modelle der chirurgischen Ausbildung, Patientenversorgung und beruflichen Prioritäten neu zu überdenken. Die Zusammenarbeit zwischen den Generationen und die proaktive Anpassung an diese Veränderungen sind unerlässlich, um eine dynamische, integrative und nachhaltige Zukunft für das Fachgebiet zu gewährleisten
			Ethische Herausforderungen durch Falschinformationen in sozialen Medien und ein Rückgang hierarchischer Mentorenbeziehungen wurden als kritische Anliegen hervorgehoben	Die Integration der Stärken der Generation Z in den Bereichen Technologie und Engagement für systemische Reformen verspricht vielversprechende Ansätze zur Bekämpfung von Burnout und zur Verbesserung der Gesundheitsversorgung	
*Satoh et al. (2025; *[9])	Umfrage	Japan	Generation Z: Wertschätzung der Arbeitszufriedenheit, Arbeit zum Wohle anderer, Bereitschaft, neue Herausforderungen anzunehmen, und Wunsch nach sofortigem Feedback	Bevorzugte Kommunikationswege der Generation Z wählen, Feedback Kultur	Japanische Ärzte der Generation Z legen Wert auf innere Motivation, wirtschaftliche Stabilität, sofortiges Feedback, digitale Technologieintegration und kollaborative Arbeitsumgebungen. Diese Erkenntnisse erfordern angepasste Ansätze in der medizinischen Ausbildung und am Arbeitsplatz, die auf die besonderen Vorstellungen der Generation Z abgestimmt sind
			Generation Z: Arbeit ist in erster Linie ein Mittel, um Geld für ihr persönliches Leben zu verdienen, bevorzugt die Arbeit im Team gegenüber der Einzelarbeit, stärkere Präferenz für proaktive Kommunikation seitens der Vorgesetzten, Chat-basierte Kommunikation am Arbeitsplatz, Zusammenarbeit statt Konkurrenz mit Kollegen, lieber mit Kollegen gleichen Alters Kontakte knüpfen		
*Schenarts (2020; *[10])	Systematische Übersichtsarbeit	USA	Historische Ereignisse und gesellschaftliche Trends sowie Veränderungen im Erziehungsstil haben zu einzigartigen Merkmalen der Generation Z geführt	Verbesserte Kommunikation und Feedbackkultur Digitale Ausbildungsprogramme, wie z. B. Internetseiten mit Ausbildungstools	Die Eigenschaften der Generation Z, wie Verantwortungsbewusstsein, Leistungsorientierung und Engagement für langfristige Ziele, unterscheiden sie von früheren Generationen
			Der bedeutendste Einfluss auf diese Generation war die weit verbreitete Nutzung von Smartphones. Dieses Gerät hat zwar ein großes Bildungspotenzial, birgt jedoch auch ein echtes Risiko, da digitale Fußabdrücke die Auswahl des Facharztausbildung und die Entwicklung von psychischen Problemen beeinflussen können	Motivation durch persönliche Beziehungen	

### Ergebnisse der Umfragen

Giunta et al. [8] führten im Oktober 2024 eine Umfrage unter 8 führenden Vertretern nationaler plastisch-chirurgischer Gesellschaften durch. Der Fragebogen bestand aus 5 offenen Fragen zu Herausforderungen, Chancen und notwendigen strukturellen Anpassungen, um den Generationenwandel in der plastischen Chirurgie aktiv zu gestalten. Es wird diskutiert, dass das Streben nach Inklusivität, Innovation und klar geregelten Arbeitszeiten der Generation Z traditionelle Ansätze in der Patientenversorgung, dem Arbeitsumfeld und der Facharztausbildung vor erhebliche Herausforderungen stellt. Allerdings bieten die Fähigkeiten dieser Generation auch Chancen, z. B. im Bereich der Digitalisierung. Die Autoren liefern konkrete Vorschläge um diesen Herausforderungen sowie Chancen aktiv zu begegnen: Einführung transparenter Kommunikationspraktiken, Integration fortschrittlicher Technologien inklusive der Nutzung künstlicher Intelligenz, Förderung der Zusammenarbeit zwischen den Generationen sowie Schaffung eines produktiven und harmonischen Arbeitsumfelds. Dabei müssen kulturelle, soziale und ökonomische Aspekte berücksichtigt werden [8].

Satoh et al. [9] führten eine Querschnittumfrage mit Schwerpunkt auf beruflichen Werten, berufsbezogenen Schulungen, der Einstellung zu Vorgesetzten und dem Arbeitsumfeld unter Ärzten im 1. bis 3. Jahr ihrer Berufstätigkeit in Japan durch. Hier arbeiteten die Kollegen vorrangig die Eigenschaften der Generation Z im Vergleich zu früheren Generationen heraus, ohne dabei wesentliche Implikationen für Ausbildung oder Arbeitsplatzattraktivität bzw. Verbesserungsvorschläge dar zu legen. Diese Eigenschaften sind: Generation Z legt Wert auf Zufriedenheit im Beruf, möchte zum Wohle anderer arbeiten, nimmt neue Herausforderungen an, wünschen sofortiges Feedback, haben eine stärkere Präferenz für proaktive Kommunikation seitens der Vorgesetzten und bevorzugen die Zusammenarbeit statt dem Wettbewerb.

### Ergebnisse der systematischen Übersichtsarbeit

Schenarts [10] gibt einen systematischen Überblick, um einen Einblick in die Generation Z zu geben, zu kontrastieren wie sie sich von früheren Generationen unterscheidet und wie man die Generation Z am Besten in einem chirurgischen Fachgebiet ausbildet. Die Generation Z betrachtet Bildung nicht nur als eine Phase der intellektuellen Stimulation, sondern vielmehr als Vorbereitung auf die berufliche Laufbahn und den finanziellen Erfolg. Die Angehörigen dieser Generation sind am ehesten bereit, sich zu engagieren, wenn sie sehen können, wie das Wissen oder die Fähigkeiten in Zukunft genutzt werden. Dabei ist Neugier der stärkste Motivator für die Wahl eines Kurses oder einer Fortbildung.

Weiterhin möchte diese Generation Bildungsziele mit eigenen Methoden erreichen, bevorzugt digitales Lernen bzw. Online-Plattformen für den Informationsgewinn, ist risikoscheu, benötigt häufigeres Feedback und wird stark motiviert durch persönliche Beziehungen sowie dem Wunsch andere nicht zu enttäuschen. Zusätzlich wird die Generation Z als leicht manipulierbar beschrieben, da sie teils objektivierbare Fakten aufgrund von Gefühlen ablehnt. Interessanterweise wird in dieser Arbeit betont, dass die Aufmerksamkeitsspanne der Generation Z nur bei sechs Minuten liegt und sogar nur bei acht Sekunden bei der Benutzung digitaler Geräte. Dies liegt deutlich unter den früheren Generationen. Laut des Autors müssen diese Charakteristika bei der Auswahl der chirurgischen Facharztkandidaten und bei der Ausbildung stringent berücksichtigt werden.

Folgerichtig arbeitet der Autor die Vor- und Nachteile der Generation Z für den chirurgischen Arbeitsmarkt heraus. Vorteile sind: Generation Z ist bereit ihren Beitrag zu leisten, legt großen Wert auf persönliche Verantwortung, möchte ethisch korrekt arbeiten, ist freiheitsliebend, sucht nach Chancen sich zu beweisen, ist nicht nur durch finanzielle Anreize motiviert und möchte Anerkennung für gute Arbeit erhalten. Dem gegenüber stehen die Nachteile: sehr kurze Aufmerksamkeitsspanne, bevorzugen digitale Kurzkommunikation (z. B. Messenger, keine E‑Mails), Probleme in der Interaktion mit anderen Generationen und haben Schwierigkeiten sich an Situationen anzupassen, in denen man auf die Entwicklung von Antworten warten muss. Außerdem können Probleme in der Interaktion/Kommunikation innerhalb ihrer eigenen Generation entstehen, da sie sich oft selbst für sehr gut halten, gleichzeitig aber sehr kritisch gegenüber Gleichaltrigen sind [10].

### Zusammenfassung: Charakterisierung der Generation Z für Ausbildung und Arbeitsmarkt

Zusammenfassend misst Generation Z der Arbeitszufriedenheit einen hohen Stellenwert bei, inklusive klar geregelter Arbeitszeiten. Sie ist bereit zum Wohle anderer mit hohem Maß an Ethik zu arbeiten und dabei gleichzeitig bereit Herausforderungen anzunehmen. Dabei hat sie ein hohes Verantwortungsbewusstsein und Engagement für langfristige Ziele. Der Wunsch nach Feedback und sofortiger Rückmeldung bzw. proaktiver Kommunikation seitens der Vorgesetzten ist essentiell. Die Generation Z bevorzugt finanzielle Sicherheit, obwohl Geld nicht der einzige Motivator ist und persönliche Beziehungen als starker Motivator gelten. Das Arbeiten im Team sowie Kooperation, insbesondere mit Gleichaltrigen, stehen über Konkurrenz und Wettbewerb am Arbeitsplatz. Digitale Lösungen für Lernen, Arbeit und Kommunikation werden bevorzugt [8–10].

Im Kontrast dazu stehen negative Aspekte der Generation Z, insbesondere die extrem kurze Aufmerksamkeitsspanne und Konflikte mit früheren Generationen [8–10].

### Zusammenfassung: Implikationen für die ärztliche Ausbildung und Arbeitsplatzattraktivität

Insgesamt gesehen muss in diesem Kontext die Digitalisierung im Bereich, Lernen, Patientenversorgung und Kommunikation schnellst möglich weiter vorangetrieben werden. Für die Generation Z muss zusätzlich ein sicheres Arbeitsumfeld mit einer guten Work-Life-Balance sowie langfristigen und nachhaltigen Perspektiven geschaffen werden. Weiterhin sollte eine regelmäßige, konstruktive Feedbackkultur etabliert sowie die intergenerationale Kommunikation gefestigt und enge persönliche Beziehungen auch am Arbeitsplatz gefördert werden.

Aktuell fehlen in allen Publikationen konkrete Pläne zur Ausgestaltung dieser Implikationen bzw. Lösungsansätze oder Strategien zur Umsetzung in den klinischen Alltag [8–10].

## Diskussion

Wir führten eine schnelle Evidenzanalyse zur Generation Z auf dem ärztlichen Arbeitsmarkt und zur Ausbildung durch, da diese Generation nun unseren aktuellen Nachwuchs darstellt. Die Beschäftigung mit dieser Thematik ist aus zwei Gründen essentiell. Zum einen benötigen wir dringend eine Steigerung der Arbeitsplatzattraktivität, um die raren Fachkräfte zum Bleiben und zur aktiven Mitgestaltung zu motivieren. Zum anderen müssen wir unsere Ausbildung stetig verbessern sowie die urologischen Standards nach aktueller Evidenzlage gewährleisten. Zwar charakterisiert diese Arbeit die Generation Z auch, doch viel wichtiger als diese Ergebnisse sind die sekundären Endpunkte, also die Implikationen für die Arbeitsplatzattraktivität und die Verbesserung der Ausbildung.

Zwar geben die Autoren der inkludierten Arbeiten Implikationen vor, wie z. B. das weitere Vorantreiben der Digitalisierung und Nutzung aller möglichen Applikationen auch für die Weiterbildung, doch fehlen in allen Publikationen konkrete Pläne zur Ausgestaltung dieser Implikationen bzw. Lösungsansätze oder Strategien zur Umsetzung im klinischen Alltag. Hier lohnt sich der Perspektivwechsel. In ihrer Arbeit zu Studierenden der Krankenpflege machen Di Mattio et al. [1] folgende Vorschläge: 1. Unterstützen Sie die Studierenden dabei, in realen Situationen mit echten Patient:innen in ihrem eigenen Tempo zu arbeiten. 2. Unterstützen Sie Studierende bei der Entscheidungsfindung und der klinischen Beurteilung durch Rollenspielszenarien, die von Mentoren und Fakultätsmitgliedern sorgfältig bewertet werden. 3. Schaffen Sie ein Lernumfeld, das sich auf Selbstfürsorge, Erfolg und Zugehörigkeit konzentriert. Diese Aspekte könnten ebenfalls für die ärztliche Ausbildung sowie das interprofessionelle Lernen zusammen mit Pflegenden interessant sein und bedürfen, unserer Einschätzung nach, weiterer Evaluation.

Eine weitere interessante Perspektive liefert auch die Arbeit von Messerer et al. [11], die sich mit der Frage beschäftigt, welche Faktoren Generation-Z-Studenten motivieren, selbst Lehrer bzw. Tutor im Medizinstudium zu werden am Beispiel eines Anatomiekurses. Dies ist ebenfalls ein wichtiger Punkt in der Ausbildung, da man als Arzt immer auch Lehrtätigkeiten übernimmt, für Patienten, um sie über die wesentlichen Aspekte ihrer Erkrankung aufzuklären, für den ärztlichen Nachwuchs sowie für den interdisziplinären Austausch oder die Weiterbildung der Pflegenden. Die größten Motivatoren, die diese Arbeit identifizieren konnte, waren die Möglichkeit das eigene anatomische Wissen zu verbessern, die Freude am Lehren sowie die Verbesserung der eignen Lehrfähigkeiten [11]. Diese Faktoren können, unserer Meinung nach, auch für die Verbesserung der urologischen Ausbildung genutzt werden, wenn man z. B. den Facharztkandidaten vermittelt, dass das Lehren immer auch die eigenen Kompetenzen verbessert.

Weiterhin veröffentlichten Singh et al. [12] im Jahr 2021 eine Umfrage unter Angehörigen der Generation der Millenials (Jahrgänge 1981 bis 1996) zu Faktoren, die die Arbeitsplatzattraktivität beeinflussen. Leider gibt es, zumindest unseres Wissens, keine Arbeiten diesbezüglich zur Generation Z. Es konnten 4 wesentliche Faktoren identifiziert werden: Praxiskultur, Work-Life-Balance, finanzielle Überlegungen sowie Karriereförderung. Dabei stellt sich die Praxiskultur als wichtigster Faktor dar, um ein Krankenhaus für die Ausbildung zu wählen und auch dort zu bleiben. Für die Generation der Millenials und ebenfalls als allgemeingültige Definition, ist Organisations- bzw. Praxiskultur die gemeinsamen Werte und Überzeugungen hinsichtlich Normen und angemessenem Verhalten in einer Organisation. Konkreter ist die Kultur für die Millenials assoziiert mit Arbeitskontrolle, Zusammenhalt, Qualität vor Produktivität, klare Kommunikation sowie die Werteausrichtung des gesamten Teams inklusive der Führungsebene [12]. Bleibt die Frage offen, ob diese Faktoren ebenfalls für die Generation Z für die Arbeitsplatzattraktivität von Bedeutung sind. Dies gilt es unbedingt zu evaluieren, da nur so eine gute Ausbildungsatmosphäre geschaffen werden kann und Mitarbeiter langfristig gebunden werden können.

Für die Generation Z muss ein sicheres Arbeitsumfeld mit einer guten Work-Life-Balance bestehen

Aus der Literatur ergeben sich aus unserer Sicht folgende Lösungsansätze in unserem Fachgebiet mit Generation Z umzugehen. Erstens müssen regelmäßige Entwicklungsgespräche geführt und eine systematische sowie konstruktive Feedbackkultur gelebt werden, so dass der Nachwuchs Planungs- und Entwicklungssicherheit hat. Zweitens muss unser Nachwuchs sichere Quellen- und Methodenkritik Erlernen, da soziale Medien und die Digitalisierung zwar enormes Potenzial haben unser Arbeitsleben zu verbessern, doch ist z. B. künstliche Intelligenz nur so gut wie die Qualität der eingegebenen Daten. Drittens der Aufbau einer freundlichen Krankenhauskultur inklusive transparenter Kommunikationswege, Integration moderner Technologien in alle Aspekte des klinischen Alltags sowie die Förderung der Zusammenarbeit zwischen den Generationen. Allerdings bedürfen all diese Lösungsansätze weiterer Forschungsarbeit und einer kontinuierlichen Evaluation mit stetiger Verbesserung sowie Anpassung. Zusätzlich könnte es notwendig werden fachspezifisch zu sein, daher sollten auch wir Urologen, insbesondere in Hinblick auf den Fachkräftemangel und der demographischen Entwicklung, aktiv an dieser Forschungsarbeit für unser Fach beteiligen.

Interessanterweise arbeiten alle Autoren stringent heraus, dass die Arbeit mit Generation Z sowohl Vor- als auch Nachteile hat, daher gilt es die Vorteile für das Arbeitsumfeld und die Weiterbildung vollkommen auszunutzen. Wichtiger in diesem Kontext scheint allerdings die Frage, wie man mit den Schwächen umgeht und auf diese reagiert. Besonders problematisch sind in diesem Zusammenhang zum einen die extrem kurze Aufmerksamkeitsspanne sowie die Problematik der Kommunikation mit anderen Generationen. Ein Lösungsansatz könnte die Entwicklung digitaler Lernprogramme für die entsprechenden Anforderungen bzgl. der Aufmerksamkeitsspanne sein, doch auch hier ist weitere Forschungsarbeit notwendig.

Zusätzlich muss noch betont werden, dass in einem operativen Fachgebiet, wie der Urologie, lange Konzentrationsphasen unerlässlich sind und ggf. auch Lernprogramme entwickelt werden müssen, um die Aufmerksamkeitsspanne zu verbessern. Unklar bleibt leider die Kernfrage, wie genau man die Zusammenarbeit/Kooperation zwischen den Generationen in der Medizin verbessert bzw. fördert? Hier könnten Umfragen und Konsensusprozesse erste Ideen sowie Ansätze liefern, die selbstverständlich in einem nächsten Schritt validiert werden müssen. Konsequenterweise plant unsere Arbeitsgruppe zur Verbesserung der urologischen Ausbildung und Nachwuchsförderung sich weiter intensiv mit dieser Thematik auseinander zu setzen. Als ersten Schritt haben wir eine schweizweite Umfrage zu Karrierewegen in der Urologie durchgeführt, deren Ergebnisse wir Ende des Jahres erwarten.

Geschlechtsspezifische Unterschiede in den Bedürfnissen bei der Ausbildung sowie am Arbeitsplatz dürfen zusätzlich nicht vernachlässigt werden, da der Anteil der Medizinstudierenden in den letzten Jahren stetig zugenommen hat. Trotz dieses Anstiegs ist der Anteil von Frauen unter den habilitierten Ärzten noch immer gering [13, 14]. Messerer et al. [11] stellten in ihrer Arbeit heraus, dass Studentinnen möglicherweise eine gezielte Berufsberatung benötigen, um dem Mangel an weiblichen Vorbildern im Berufsleben entgegenzuwirken. Es gibt also ebenfalls möglicherweise geschlechtsspezifische Unterschiede in Hinblick auf Bedürfnisse bei Arbeitsplatzattraktivität und ärztlicher Ausbildung. Dies sollte zukünftig herausgearbeitet und ggf. evaluiert werden. Daten aus der Urologie fehlen hier leider ebenfalls.

Leider ist auch diese Arbeit nicht ohne Limitationen, insbesondere da es sich um eine schnelle Evidenzanalyse mit der Nutzung nur einer Datenbank handelt und es damit zu einem Selektionsbias gekommen sein kann. Weiterhin kommen die inkludierten Arbeiten mehrheitlich nicht aus dem deutsch-sprachigen Raum und es muss diskutiert werden, dass es bzgl. der Arbeitsplatzattraktivität sowie auch bei der Ausbildung auch kulturelle Aspekte ins Gewicht fallen bzw. berücksichtigt werden müssen. Weiterhin fehlt leider Evidenz aus dem Fachgebiet der Urologie somit können aus dieser Arbeit heraus auch keine konkreteren Vorschläge für Ausbildungsprogramme gemacht werden, doch Ziel dieser Arbeit ist es auch für die Thematik zu sensibilisieren.

Die Arbeit liefert erste Implikationen für die Ausbildungsverbesserung der Generation Z

Trotzdem liefert unsere Arbeit erste Implikationen für die Ausbildungsverbesserung der Generation Z. Allerdings müssen entsprechende Programme entwickelt und validiert werden. Dies ist aber in Hinblick auf den Fachkräftemangel sowie die demographische Entwicklung, von der die Urologie besonders stark betroffen ist, von essentieller Bedeutung. Weiterhin ist es für Arbeitgeber wichtig, attraktive Arbeitsplätze zu bieten und hochqualifizierte Fachkräfte langfristig zu binden, auch zu diesem Aspekt liefert unsere Arbeit erste Daten.

## Fazit für die Praxis

- Die Generation Z hat bzgl. der Ausbildung und Arbeitsplatzattraktivität andere Bedürfnisse als frühere Generationen.
- Für die Generation Z muss ein sicheres Arbeitsumfeld mit einer guten Work-Life-Balance sowie langfristigen und nachhaltigen Perspektiven geschaffen werden.
- Die Integration moderner Technologien in den Arbeitsalltag ist essentiell.
- Probleme der Generation Z sind vorrangig die kurze Aufmerksamkeitsspanne sowie die Kommunikation mit früheren Generationen.
- Aufgrund des Fachkräftemangels und der demographischen Entwicklung ist die Neugestaltung von Ausbildungsprogrammen für die Generation Z und deren Evaluation sowie stetige Verbesserung dringend erforderlich.
- Weitere Untersuchungen und Evaluation von Ausbildungsprogrammen spezifisch für die Generation Z im Fachgebiet der Urologie sind dringend erforderlich.
